# Enhancement of fiber content and cannabinoids of hemp using arbuscular mycorrhizal fungi and endophytic fungi

**DOI:** 10.1038/s41598-026-46869-0

**Published:** 2026-04-02

**Authors:** Wasan Seemakram, Jakkapat Paluka, Waroon Khota, Thanapat Suebrasri, Chanon Lapjit, Thomas W. Kuyper, Sophon Boonlue

**Affiliations:** 1 Department of Microbiology and Parasitology, Faculty of Medical Science, Naresuan University, Phitsanulok, 65000 Thailand; 2 Research Administration Division, Khon Kaen University, Khon Kaen, 40002 Thailand; 3 Faculty of Natural Resources, Rajamangala University of Technology Isan, Sakon Nakhon, 47160 Thailand; 4 Division of Basic and Preclinical Sciences, Institute of Science and General Education, Nakhon Ratchasima College, Nakhon Ratchasima, 30000 Thailand; 5 Cannabis Research Institute, Khon Kaen University, Khon Kaen, 40002 Thailand; 6 Department of Horticulture, Faculty of Agriculture, Khon Kaen University, Khon Kaen, 40002 Thailand; 7 Soil Biology Group, Wageningen University & Research, P.O. Box 47, Wageningen, 6700 AA Netherlands; 8 Department of Microbiology, Faculty of Science, Khon Kaen University, Khon Kaen, 40002 Thailand

**Keywords:** *Cannabis sativa* RPF3, Hemp fiber, Organic agriculture, Plant growth promotion, Rhizosphere, Biochemistry, Biotechnology, Microbiology, Molecular biology

## Abstract

This study aimed to investigate the efficiency of arbuscular mycorrhizal fungi (AMF) and endophytic fungi to promote growth and fiber yield of *Cannabis sativa* subsp. *sativa* RPF3 (Hemp) and the impact on cannabinoid concentrations. A factorial pot experiment with six replications was conducted for 90 days. Two species of AMF (*Rhizophagus aggregatus*,* R. prolifer*) and two species of endophytic fungi (*Lasiodiplodia theobromae*,* Macrophomina phaseolina*) were selected as inocula and compared with two non-mycorrhizal controls, one without synthetic fertilizer and one with synthetic NPK fertilizer. Inoculation with AMF and endophytic fungi increased mass fractions of cellulose, acid detergent fiber, and neutral detergent fiber and mass fractions of cannabinoids, especially of cannabidiol, in leaves and shoots, with the strongest increase noted after inoculation with *R. aggregatus* and *L. theobromae*. Our study is the first to report the effectiveness of AMF and endophytic fungi on promoting growth, fiber content, and cannabinoid production in hemp. These results suggest the potential for hemp cultivation with AMF and endophytic fungi without the potential negative effects induced by high use of synthetic fertilizer.

## Introduction

Hemp (*Cannabis sativa* subsp. *sativa*) is an annual herbaceous plant belonging to the Cannabaceae family that is the same family as marijuana. Although hemp is closely related to marijuana, their physical and chemical characteristics are different. In this regard, the hemp plant is taller, and the leaves are slenderer and lighter in color than those of marijuana. Hemp as a fiber crop has high fiber yield and quality, while containing (much) lower levels of tetrahydrocannabinol (THC) and cannabidiol (CBD) than marijuana^1^. Hemp fiber consists of two types: bast fiber and shives (woody core). Bast fibers are considered as the high-quality fiber, possessing strongest and stiffest fiber among all vegetable fibers^2^. Chemically, bast fibers of hemp are composed of 70–74% cellulose, 15–20% hemicellulose, and 3–6% lignin^3^. It is not only a high-yielding fiber crop that can meet the high global demand for fiber, but also is a raw material for a diverse range of products, e.g., agriculture, textile, papermaking, automotive, construction, functional food, oil, cosmetics, personal care, and pharmaceutical products^4^. Hemp as a renewable and sustainable natural material is regaining its popularity from all over the world. Hemp spread globally from its origin in Central Asia, due to its adaptability to various climate conditions and soil properties. Farm practices like plant density, use of synthetic fertilizers, harvesting time, and climatic conditions are very important for fiber yield and quality development of fiber^5^. Accordingly, improving plant growth and fiber content has gained more attention. The most common and easily accessible practice is the use of synthetic fertilizer to enhance plant production. However, undesirable effects of overuse of synthetic fertilizer and toxic chemical residues have been observed after long-term use, resulting in lower soil quality, notably a reduction of beneficial soil microbes^6^. In order to reduce the use of synthetic fertilizers while promoting the growth and yield of hemp, plant growth promoting microorganisms (PGPM) are of interest and they have been extensively studied due to their environmentally friendly nature^7^.

Arbuscular mycorrhizal fungi (AMF), belonging to the Subphylum Glomeromycotina^8^, establish a mutualistic symbiosis with plant roots. AMF occur in nearly all types of ecosystems and are formed naturally by most plant species. AMF develop extraradical mycelia that extend the depletion zone that develops around roots and facilitate the acquisition of nutrients of low mobility^9^. AMF have beneficial effects on nutrient uptake, such as phosphorus (P), nitrogen (N), potassium (K), and micronutrients resulting in enhanced plant growth^10^. AMF can also help plants to grow under unfavorable environmental conditions, such as arid conditions, and prevent plant diseases in the root system^9^. Endophytic fungi, mostly belonging to the phylum Ascomycota, have been of interest because they reside within plant tissues or organs without causing any visible signs of infection^11^. Endophytic fungi equally can play an essential role in plant growth promotion, leading to a higher yield by an increase of nutrient uptake, reduction in disease severity, improvement of host resistance against environmental stresses, and production of secondary metabolites (phenols and flavonoids)^12^. Moreover, endophytic fungi play a role in enhancing the capability of host plants to produce plant growth hormones such as auxin, gibberellins, cytokinin, abscisic acid, and in particular, indole-3-acetic acid (IAA) and gibberellic acid (GA3)^13^.

Both hemp and marijuana are colonized by AMF. Zielonka et al.^14^ showed the presence of several AMF species associated with industrial hemp, such as *Funneliformis mosseae*, *F. caledonium*, and *F. geosporum*. Kakabouki et al.^15^ demonstrated beneficial effects of *Rhizophagus irregularis* on hemp, whereas Seemakram et al.^6^ reported beneficial effects of *R. aggregatus* and *R. prolifer*. There are also reports on the occurrence of endophytic fungi in hemp. Kusari et al.^16^ focused on aboveground endophytes and their potential as biocontrol agents against plant diseases. Other studies looked at the fungal microbiome in rhizosphere soils and in roots^17^. Due to the forbidden nature of *C. sativa* in the past, scientific research on microbial diversity in cannabis fields is still largely unknown^18^. Studies on AMF and endophytic fungi demonstrated enhanced production of secondary compounds like cannabinoids in *C. sativa*, and this effect would not necessarily be beneficial for the cultivation of industrial hemp^6^. It is therefore relevant to assess the simultaneous effects of AMF and endophytic fungi on various aspects of hemp performance, including biomass, fiber concentrations, and concentrations of cannabinoids.

In this study, two different species of AMF, *Rhizophagus aggregatus* and *R. prolifer* were selected to test their ability to promote plant growth, yield, and accumulation of secondary metabolites. In addition, since there are still no reports on effects of endophytic fungi on fiber production and secondary metabolite accumulation in hemp, we also isolated endophytic fungi from hemp and investigated their plant growth promoting properties. The results of this study could provide insights into application of both AMF and endophytic fungi for promoting eco-friendly cultivation of hemp in Thailand.

## Results

### Isolation and identification of endophytic fungi

Two fungal isolates (WS-TS-A1; SS1R1) were selected for further experimentation due to their efficient plant growth promoting properties. These isolates were identified using morphological characteristics and molecular approaches. The results indicated that the ITS and TUB2 gene sequences of isolate WS-TS-A1 were 100% identical to the sequence of *Lasiodiplodia theobromae* strain C-38; MFLUCC 24–0212 from Thailand (Accession Numbers PP960254 and PP982562 for ITS and TUB2 respectively)^19^, while the ITS, TEF1-α and CaM gene sequence of isolate SS1R1 was 100% identical to the sequence of *Macrophomina phaseolina* MPK:01 MT (Accession Number MT186826)^20^. Therefore, isolate WS-TS-A1 and isolate SS1R1 were designated as *Lasiodiplodia theobromae* (ITS, Accession number LC746163; TUB2, Accession number PP793903) and *Macrophomina phaseolina* (ITS, Accession number LC746163; TEF1-α, Accession number PP793904; CaM, Accession number PP793905), respectively throughout this study.

### Pathogenicity of endophytic fungi

**Fig. 1:**
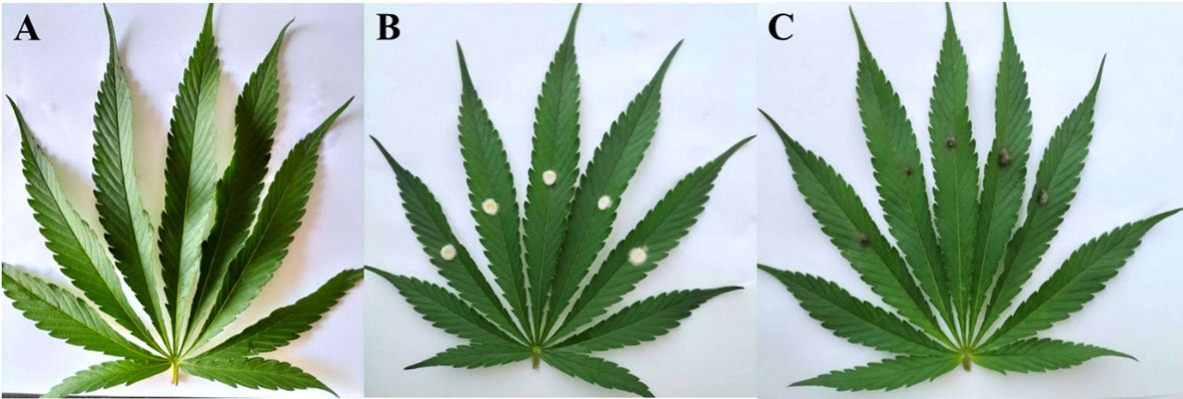
Pathogenicity test of endophytic fungal isolates on healthy hemp leaves: control uninoculated (A) and leaves inoculated with *Macrophomina phaseolina* (B), and *Diplodia theobromae* (C).

Both isolates did not produce necrotic lesions on hemp plants (Fig. 1). The same fungal isolates were re-isolated from inoculated leaves to confirm Koch’s postulates. Both isolates are apparently not pathogenic to cannabis plants under our experimental conditions.

### Characterization of plant growth promotion by endophytic fungi

Both endophytic fungal species produced IAA under conditions with and without tryptophan (Table 1). The concentration of IAA was higher in *M. phaseolina* than in *L. theobromae* and higher in the presence than in the absence of tryptophan. Likewise, *M. phaseolina* produced more GA3 than *L. theobromae*. *Lasioplodia theobromae* produced more ammonia than *M. phaseolina*. Similarly, *L. theobromae* was more efficient in solubilizing phosphate and had higher acid and alkaline phosphatase levels than *M. phaseolina*. These results suggest that both endophytic fungal strains are effective plant growth promoters, but with different modes of action.

**Table 1:** Plant growth promoting properties of both endophytic fungal species; ± refers to standard error.

Isolates	IAA without L-tryptophan (ppm)	IAA with L-tryptophan (ppm)	GA_3_ production (ppm)	Ammonia production (ppm)	Phosphate solubilization (ppm)	Acid phosphatase (U/mL)	Alkaline phosphatase (U/mL)
*L. theobromae*	21.6 ± 0.1b	58.9 ± 0.1b	450 ± 0.3b	187.4 ± 0.9a	1405 ± 2.0a	29.5 ± 0.2a	2.8 ± 0.01a
*M. phaseolina*	29.5 ± 0.1a	69.8 ± 0.1a	553 ± 0.2a	27.5 ± 0.1b	685 ± 1.0b	10.5 ± 0.1b	0.5 ± 0.02b
%CV.	10	4	11	12	9	5	5
F-Test	*	**	*	*	**	**	**

### Aboveground plant performance

**Fig. 2:**
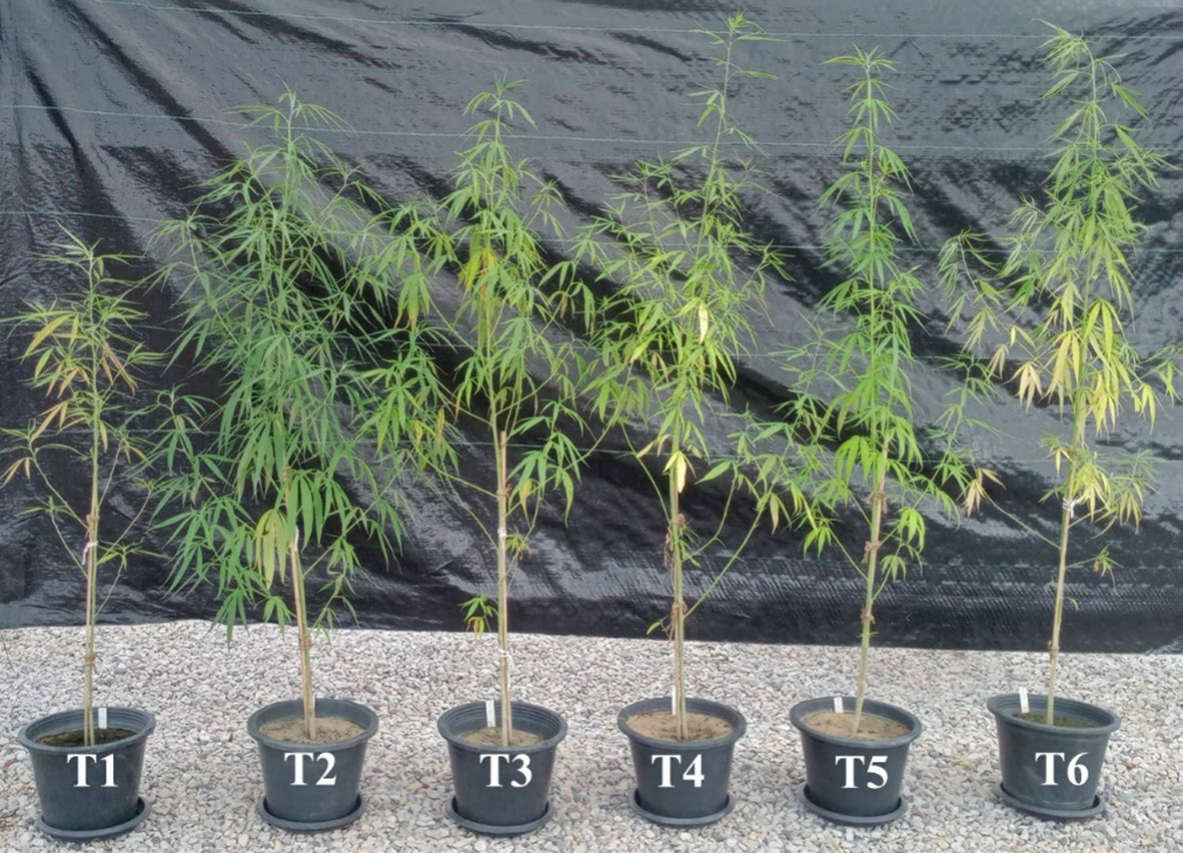
Morphologies of *Cannabis sativa* RPF3 at the harvest stage (90 days) grown under different conditions: T1, control without AMF inoculum, without synthetic fertilizer; T2 Control with synthetic NPK fertilizer; T3, Plants inoculated with *R. prolifer*; T4, Plants inoculated with *R. aggregatus*; T5, Plants inoculated with *L. theobromae* and T6, Plants inoculated with *M. phaseolina*.

All aboveground plant performance parameters of the inoculated plants (T3-T6) were higher than those of the non-inoculated plants (T1), regardless of types and species of the inoculated microorganisms (Table 2). Control plants with synthetic fertilizer (T2) had the highest SPAD, leaf area index, and leaf dry weight, which were significantly higher than those of the other treatments. Although the effects of the inoculation with either AMF or endophytic fungi on plant growth promotion were not superior to those of the synthetic fertilizer, inoculation with *M. phaseolina* enhanced plant growth parameters to the level comparable to plants treated with synthetic fertilizer. This result suggests that *M. phaseolina* inoculum is potentially to be of use in place of synthetic fertilizer for enhancing hemp growth. The effects of inoculation with AMF (T3-T4) were comparable to those of inoculation with endophytic fungi (T5-T6). Apparently, both AMF and endophytic fungi could enhance growth and biomass of hemp in a relatively similar manner even though both fungal groups possess different PGP properties. The morphologies of cannabis plants in each treatment at harvest stage are shown in Fig. 2.

**Table 2:** Aboveground plant performance parameters of *Cannabis sativa* RPF3 grown under different conditions at harvest stage.

Treatment	SPAD	Height (cm)	Diameter (mm)	Leaf area index (cm^2^)	Leaf dry weight (g)	Shoot dry weight (g)	Stem dry weight (g)	Bast-fibers dry weight (g)
T1	38.2 ± 0.1c	120 ± 1b	10.4 ± 0.1c	1511 ± 10c	7.7 ± 0.2c	1.8 ± 0.1c	17.1 ± 0.3c	2.8 ± 0.1b
T2	54.2 ± 0.1a	159 ± 2ab	14.2 ± 0.1a	6234 ± 12a	29.8 ± 0.1a	7.6 ± 0.2a	31.8 ± 0.2a	4.9 ± 0.1a
T3	44.3bc	178 ± 1a	12.0 ± 0.1bc	2633 ± 14b	12.7 ± 0.3bc	3.0 ± 0.1b	23.1 ± 0.1abc	4.9 ± 0.1a
T4	41.8 ± 0.1bc	172 ± 5a	11.0 ± 0.1bc	2257 ± 11bc	10.8 ± 0.5bc	2.6 ± 0.2b	21.9 ± 0.4bc	4.3 ± 0.2ab
T5	42.1 ± 0.1bc	177 ± 4a	12.1 ± 0.1bc	2275 ± 13bc	13.1 ± 0.3bc	4.2 ± 0.4ab	28.3 ± 0.1ab	4.6 ± 0.1ab
T6	47.1b	174 ± 4a	12.7 ± 0.1ab	2845 ± 12b	15.8 ± 0.1b	5.9 ± 0.4a	24.7 ± 0.1abc	5.5 ± 0.4a
%CV.	10	13	11	19	18	19	25	18
F-Test	**	**	**	**	**	*	*	**

Numbers followed by the same letter in each column indicate data which were not significantly different according to LSD test, F-test: * Significant difference at *P* ≤ 0.05, ** Significant difference at *P* ≤ 0.01; (T1: control without microbial inoculum, T2: Synthetic fertilizer, T3: *R. prolifer*, T4: *R. aggregatus*, T5: *L. theobromae* and T6: *M. phaseolina.*

### Root traits

Most root traits were not significantly affected by the inoculation of either AMF or endophytic fungi, compared with the unfertilized control plant (Table 3). Nevertheless, root length and surface area of the plants inoculated with *R. prolifer* was significantly higher than those of the control plants, while they were not different from those of the plants treated with synthetic fertilizer. Likewise, root dry weight of the inoculated plants was comparable to that of the control plants treated with synthetic fertilizer, and both were also significantly higher than that of the unfertilized control plant. Even though inoculation with *L. theobromae* did not have much impact on the root traits, it enhanced root tissue density compared with plants in the other treatments.

**Table 3:** Root properties and leaf nutrient concentrations of the cannabis plants grown under different conditions.

Treatment	Length (m)	Surface area (cm^2^)	Average diameter (mm)	Root dry weight (g)	Specific root length (m g^− 1^)	Root tissue density (g cm^− 3^)	*N* concentration (mg g^− 1^)	*P* concentration (mg g^− 1^)	K concentration (mg g^− 1^)
T1	292 ± 81c	3009 ± 56c	0.28 ± 0.05a	6.0 ± 1.8b	55 ± 2.8a	0.20 ± 0.05a	5.6 ± 0.2c	0.5 ± 0.03c	7.4 ± 0.2c
T2	491 ± 47a	4343 ± 56a	0.31 ± 0.05a	10.5 ± 2.9a	47 ± 0.8a	0.18 ± 0.06a	20.8 ± 0.4a	3.3 ± 0.05a	17.0 ± 0.3a
T3	422 ± 61ab	3912 ± 86ab	0.29 ± 0.02a	9.3 ± 1.2ab	46 ± 0.5a	0.20 ± 0.05a	10.4 ± 0.2ab	1.6 ± 0.04b	10.8 ± 0.2abc
T4	402 ± 72abc	3339 ± 48bc	0.32 ± 0.08a	8.5 ± 1.5ab	47 ± 0.5a	0.18 ± 0.06a	8.2 ± 0.1b	1.0 ± 0.08b	7.4 ± 0.6c
T5	345 ± 74bc	3019 ± 60c	0.28 ± 0.03a	9.7 ± 0.8ab	32 ± 0.7a	0.29 ± 0.01a	7.5 ± 0.3b	1.0 ± 0.01b	9.9 ± 0.1bc
T6	365 ± 21abc	3656 ± 61abc	0.32 ± 0.03a	10.5 ± 0.8ab	35 ± 0.2a	0.22 ± 0.03a	7.5 ± 0.2b	1.30.03b	14.2 ± 0.6ab
%CV.	22	16	17	18	29	28	25	20	23
F-Test	*	*	n.s.	*	ns.	ns.	**	**	*

Numbers followed by the same letters in each column indicate the data which were not significantly different according to LSD test, * Significant difference at *P* ≤ 0.05, ** Significant difference at *P* ≤ 0.01; (T1: control without fertilizer, T2: control with mineral fertilizer, T3: *R. prolifer*, T4: *R. aggregatus*, T5: *L. theobromae* and T6: *M. phaseolina.*

### Aboveground plant nutrient concentrations

Inoculated, but unfertilized plants, either with AMF or endophytic fungi significantly enhanced N and P concentrations compared with the unfertilized control plant (Table 3). The K concentration was enhanced only in the treatment with *R. prolifer* and *M. phaseolina*. Plants treated with synthetic fertilizer showed a significantly higher nutrient (N, P, K) concentration than plants in the other treatments.

### Root colonization and spore density

**Fig. 3:**
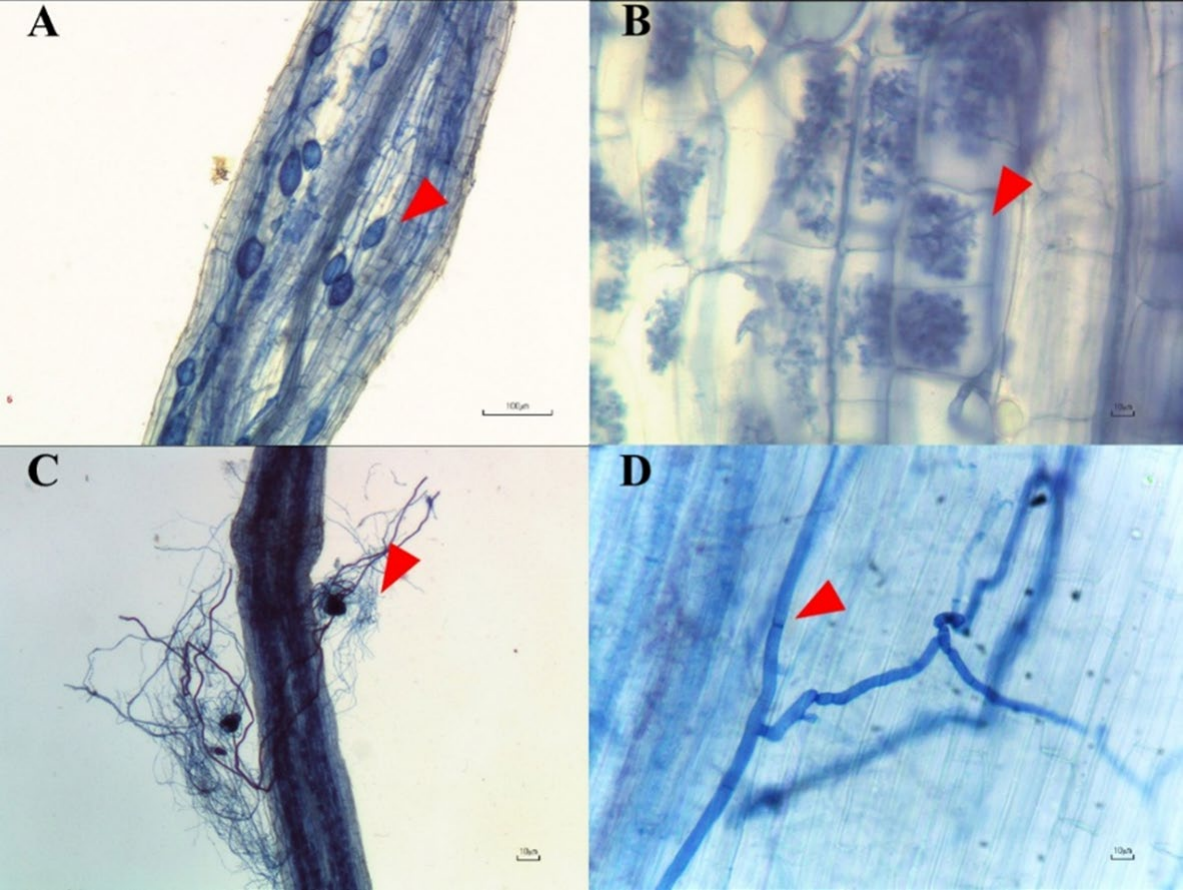
The effects of AMF and endophytic fungi on colonization in root hairs of *Cannabis sativa*. A), intensity of vesicles (arrow head); B), Arum-type of arbuscules (arrow head); C), AMF hyphae and spore around plant roots (arrow head) and D), Hyphae of endophytic fungi (arrow head).

Mycorrhizal fungal spores and fungal structures such as hyphae, arbuscules, and vesicles were observed only in pots of both mycorrhizal treatments (T3, T4). In contrast, only hyphae were present in pots without AMF inoculum but with endophytic fungal inoculum (T5, T6). The fractional root colonization ratio was not significantly different between pots inoculated with AMF and those inoculated with endophytic fungi (*R. aggregatus* – 26%; *R. prolifer* – 17%; *L. theobromae* – 17%; *M. phaseolina* – 14%). These results indicated that both AMF and endophytic fungi successfully colonized roots of hemp plants (Fig. 3).

### Fiber concentrations

Plants that were inoculated with either AMF or endophytic fungi had fiber contents comparable to those of plants treated with synthetic fertilizer, and even higher in some cases, all of which being significantly higher than that of the control plants without synthetic fertilizer (Table 4). The highest NDF and ADF concentrations (70.5% and 58.5% respectively) were found in plants inoculated with *R. aggregatus*. Likewise, relatively high concentrations of ADL were found in plants inoculated with *R. aggregatus* and with *L. theobromae*, although they were not different from those of plants treated with synthetic fertilizer. Inoculation with AMF led to higher concentrations of hemicellulose and cellulose than inoculation with endophytic fungi (Table 5). These results imply the potential application of microbial inocula to enhance growth and yield of hemp without synthetic fertilizer.

**Table 4:** Contents of fibers produced by *Cannabis sativa* RPF3 grown under different conditions at the harvest stage.

Treatment	NDF (%)	ADF (%)	ADL (%)	Cellulose (%)	Hemicellulose (%)
T1	60.5 ± 2.0c	50.2 ± 2.0c	3.3 ± 0.3bc	46.9 ± 2.0c	10.3 ± 1.0ab
T2	63.7 ± 1.0b	54.6 ± 1.0b	4.0 ± 0.2ab	50.6 ± 1.0b	9.1 ± 2.0b
T3	66.4±.0b	53.7 ± 3.0b	2.5 ± 0.2c	51.2 ± 1ab	12.7 ± 1.0a
T4	70.5 ± 3.0a	58.5 ± 3.0a	4.2 ± 0.3ab	54.3 ± 4.0a	12.0 ± 1.0ab
T5	67.0 ± 2.0b	55.3 ± 2.0b	4.9 ± 1.0a	50.4 ± 2.0b	10.7 ± 2.0ab
T6	63.5 ± 1.0bc	52.8 ± 1.0bc	3.1 ± 0.2bc	49.7 ± 3.0b	10.7 ± 1.0ab
%CV.	3	3	19	3	18
F-test	**	*	**	*	**

Numbers followed by the same letters in each column indicate data that were not significantly different according to LSD test, * Significant difference at *P* ≤ 0.05, ** Significant difference at *P* ≤ 0.01; (T1: control without microbial inoculum and synthetic fertilizer, T2: control with synthetic fertilizer, T3: *R. prolifer*, T4: *R. aggregatus*, T5: *L. theobromae* and T6: *M. phaseolina*.

**Table 5:** Concentrations of cannabinoids produced by *Cannabis sativa* RPF3 grown under different conditions.

Treatments	Concentration in dry sample (mg g-1)				Total of cannabinoids (g)			
	CBD		THC		CBD		THC	
	Leaf	Shoot	Leaf	Shoot	Leaf	Shoot	Leaf	Shoot
T1	0.63 ± 0.02d	0.03 ± 0.02a	0.78 ± 0.04a	0.04 ± 0.01a	4.83 ± 0.3e	0.24 ± 0.01c	1.40 ± 0.4a	0.07 ± 0.01b
T2	0.62 ± 0.03d	0.03 ± 0.03a	0.63 ± 0.06a	0.03 ± 0.01a	18.32 ± 0.1a	0.96 ± 0.02a	4.78 ± 0.1a	0.24 ± 0.01a
T3	0.99 ± 0.01b	0.03 ± 0.01a	0.70 ± 0.02a	0.03 ± 0.02a	12.56 ± 0.1c	0.41 ± 0.05bc	2.09 ± 0.2a	0.10 ± 0.03ab
T4	0.92 ± 0.01bc	0.04 ± 0.01a	0.70 ± 0.03a	0.03 ± 0.01a	9.89 ± 0.2d	0.43 ± 0.03cb	1.86 ± 0.2a	0.08 ± 0.01b
T5	1.19 ± 0.03a	0.05 ± 0.01a	0.56 ± 0.05a	0.03 ± 0.01a	15.54 ± 0.3b	0.69 ± 0.02ab	2.35 ± 0.1a	0.12 ± 0.02ab
T6	0.82 ± 0.01c	0.04 ± 0.01a	0.53 ± 0.07a	0.02 ± 0.02a	13.04 ± 0.01c	0.62 ± 0.01b	3.11 ± 0.1a	0.14 ± 0.01ab
%CV.	8	26	22	30	7	22	27	15
F-Test	**	ns	ns	ns	**	*	ns	*

Numbers followed by the same letters in each column indicate data that were not significantly different according to LSD test, * Significant difference at *P* ≤ 0.05, ** Significant difference at *P* ≤ 0.01; (T1: control without microbial inoculum and synthetic fertilizer, T2: control with synthetic fertilizer, T3: *R. prolifer*, T4: *R. aggregatus*, T5: *L. theobromae* and T6: *M. phaseolina*.

### Cannabinoid concentrations

**Fig. 4:**
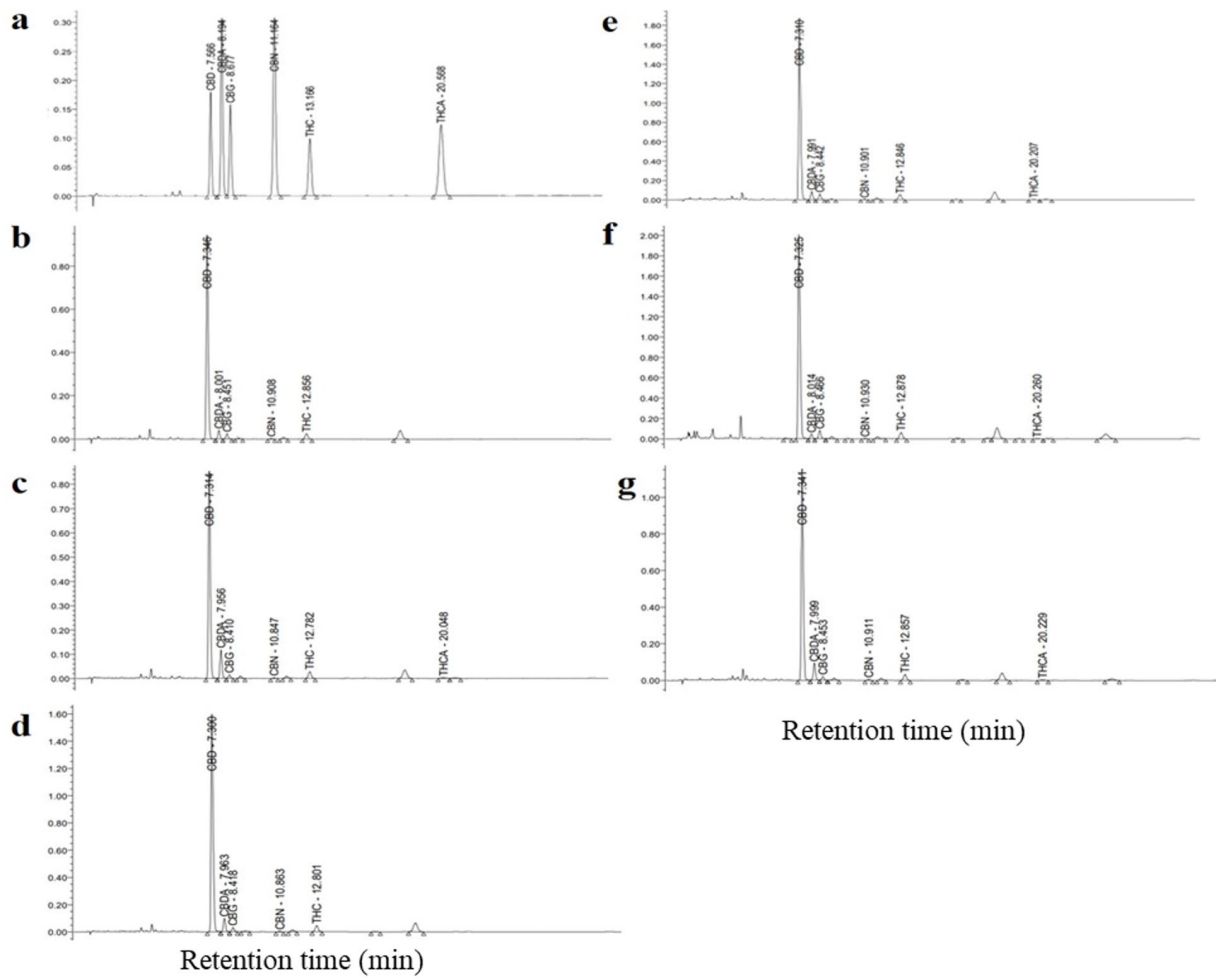
Identification of cannabinoids treated with endophytic fungi and AMF using high-performance liquid chromatography. HPLC chromatograms are indicated as follows: a); Cannabinoids compound standard, b); without AMF and endophytic fungi, c); without AMF, with synthetic fertilizer, d); crude extract of *R. prolifer*, e); crude extract of *R. aggregatus*, f); crude extract of *L. theobromae* and g, crude extract of *M. phaseolina*. Cannabinoids are abbreviated as follows: Cannabidiolic (CBD), Tetrahydrocannabinolic (THC), Cannabinol (CBN), Cannabidiolic acid (CBDA), Tetrahydrocannabinolic acid (THCA) and Cannabigerolic (CBG). Each chromatogram is a representative of triplicate analysis.

HPLC chromatograms (Fig. 4) showed that plants in all treatment produced both CBD and THC, while no CBDA or THCA was detected due to the decarboxylation of CBDA into CBD, and THCA into THC during sample drying by heating at 100 °C for 3 h. The amount of each type of cannabinoids produced by plants grown under different treatments is shown in Table 5. Both inoculation with AMF and with endophytic fungi significantly enhanced CBD and THC concentrations in hemp plants compared with the controls. Concentrations of CBD and THC were lowest in non-fertilized, non-mycorrhizal plants, and highest in plants inoculated with *R. aggregatus* and *L. theobromae*, while plants that received synthetic fertilizer had intermediate concentrations, lower than those found in plants inoculated with AMF and endophytic fungi. However, because of the large effects of synthetic fertilizer on plant biomass, plants fertilized with synthetic fertilizer had the highest amounts of CBD and THC. Plants inoculated with endophytic fungi had higher CBD and THC contents than plants inoculated with AMF, an effect largely due to different impacts of both fungal guilds on plant performance and biomass (Table 4).

## Discussion

In this study, we molecularly identified two fungal endophyte species as *Lasiodiplodia theobromae* and *Macrophomina phaseolina*. Generally, fungi of the genera *Lasiodiplodia* and *Microphomina* are considered pathogenic that are commonly found in tropical and subtropical areas^21^. *Lasiodiplodia* sp. cause several diseases in many plant species for instance, canker and leaf blight in rubber trees, and fruit rot in longan^22^. *M. phaseolina* has also been reported to cause leaf blight on spider lilies in Malaysia^23^. It is a well-known plant pathogen causing several important diseases such as charcoal rot, Ashy stem blight^24^, and leaf blight^25^ etc. There are also reports of pathogenic behavior of both fungal endophyte genera on *C. sativa*. Goodnight et al.^26^ reported charcoal rot on hemp caused by *M. phaseolina* in the United States. Roberts and Punja^27^ reported stem lesions on marijuana caused by *L. theobromae*. Beneficial effects of *L. theobromae* on *C. sativa* performance have been reported before by Seemakram et al.^28^ and plant growth promotion by *Macrophomina phaseolina* has been reported before by Suebrasri et al.^29^. We inoculated leaves of hemp with these species and recovered them afterwards, thereby fulfilling Koch’s postulates. Both species did not cause harm on cannabis under our experimental conditions, suggesting they could be potentially beneficial for hemp cultivation. However, in view of the literature cited above, it would be important to extend studies on potential pathogenic effects on hemp by those two fungal endophytes. As noted by Salvatore et al.^30^ there is a thin line between endophytic growth and pathogenic behavior of *L. theobromae*, whereas Huertas et al.^31^ noted plant growth promoting effects of several strains of *M. phaesolina* under drought and salinity stress.

Our study showed that *L. theobromae* produced relatively high amounts of ammonia and dissolved phosphate, compared with other endophytes. For example, *Daldinia eschscholtzii*, *M. phaseolina*, *Trichoderma erinaceum* produced ammonia of approximately 29–75 µg mL^− 1^^29^, while *L. theobromae* in this study produced ammonia of 187 µg mL^− 1^. In addition, *Glomerella cingulata*, exhibiting high plant growth promoting ability, produced ammonia of 95 µg mL^− 1^^13^. Ammonia probably supplies nitrogen and also promotes shoot and root elongation, which subsequently increases plant biomass^32^. In addition, it can serve as a triggering factor in the suppression of plant pathogens^33^. Both endophytic fungi produced alkaline and acid phosphatases. The phosphatases are involved in organic-phosphate degradation resulting in release of orthophosphate that can subsequently become available for plant uptake and utilization^34^. According to our previous studies, both AMF species *Rhizophagus prolifer* and *R. aggregatus* enhanced plant growth in a wide range of plant types including hemp^6,9,35^. Therefore, both inoculation with AMF and endophytic fungi on hemp were expected to play an important role in improving growth and fiber production of hemp.

Inoculation with either AMF or endophytic fungi promoted the SPAD, height, stem diameter, and leaf area of the hemp. In the absence of inoculation, unfertilized control plants performed poorest, but control plants in combination with synthetic fertilizer produced the best plant performance. Inoculation with *M. phaseolina* had an effect comparable with synthetic-fertilizer application. Our results are consistent with a previous study by Suebrasri et al.^29^, who reported that another strain of *M. phaseolina* promoted plant performance of sunchoke plants. Moreover, Mutumba et al.^36^ documented that AMF had multiple beneficial effects on plant growth and development of high chlorophyll index. In addition, our results agree with previous literature on other plant species. *Rhizophagus intraradices* stimulated white clover (*Trifolium repens*) growth^37^. *Rhizophagus* spp. play an important role in promoting growth of many types of plants due to their ability to increase plant photosynthesis^15,38^.

Due to the current high interest on hemp, scientists have focused on the development of new hemp cultivation methods in relation to the purpose of the production. The cultivation process and environment strongly affect to fiber quality. Also, an increase in bast-fiber content is desirable for cellulose-rich and low-lignified long fibers. Hemp possesses primary and secondary fibers. Primary fibers are used for textile production. The count of the primary fibers does not change during the growing period of the plant; however, the fiber length increases with the increasing distance between internodes^39^. We found a higher fiber content when hemp was inoculated with *R. aggregatus.* In addition, the fraction of cellulose was also highest in that treatment.

Most cannabinoid compounds accumulate within the buds and flowers of cannabis^40^, whereas *C. sativa* RPF3 is a strain of hemp developed for the benefit of the textile industry, due to its high fiber but low cannabinoids concentrations^41^. Our specific hemp variety (RPF3) is used by Hmong people who live in the northern of Thailand to make clothes as substitute for synthetic fabric. It is characterized by low secondary-metabolite concentrations while also having a high fiber content. In our study concentrations of CBD and THC after inoculation with species of AMF were thirty-fold and threefold lower (CBD: 0.9-1.0 mg g^− 1^; THC: 0.7 mg g^− 1^) compared with a study on marijuana by Seemakram et al.^6^ whose used the same species of AMF (CBD: 31.7–32.3 mg g^− 1^; THC 1.5–1.7 mg g^− 1^). Higher amounts of cannabinoids were found in plants inoculated with endophytic fungi than in plants inoculated with AMF, although still much lower than in marijuana. The amount of CBD was highest in the treatment with *L. theobromae*. Our study is the first to show that *Lasiodiplodia* cannot only promote growth, but also effectively increase cannabinoid content in hemp. Other endophytic fungi, e.g., *Trichoderma harzianum*, can also increase CBD content in hemp compared with the control^42^.

Increased concentrations of cannabinoids after inoculation with AMF and endophytic fungi could be caused by both an enhanced nutritional status and by upregulation of the plant defense system caused by these beneficial fungi. Nutrient concentrations in cannabis have a large effect on the total amount of cannabinoid compounds^6^. Gorelick and Bernstein^43^ reported that increasing mineral-nutrient concentrations such as Ca, Mg, N and K led to an increase in CBD and THC concentrations. Plants fertilized with NPK in our study had higher leaf concentrations of N and P than unfertilized control plants; however, they did not differ in CBD and THC concentrations. Because of the large effect of synthetic fertilizer on plant biomass, the total amounts of CBD and THC were highest in fertilized control plants. Avio et al.^44^ listed a number of plant species from different families where inoculation with AMF both increased plant biomass and concentrations of bio-active compounds with differential synthesis of bio-active compounds in mycorrhizal and non-mycorrhizal plants. Their study pointed towards links between the expression of these compounds and the mycorrhizal role in priming plant defense responses against pathogens. More specifically, they suggested a major role for plant hormones such as jasmonic acid and abscisic acid. Next to a role of AMF in regulating plant hormones, a further role for IAA and GA_3_ could be suggested based on studies on endophytic fungi, as IAA and GA_3_ could modulate shoot branching^13^.

## Methods

### Isolation and identification of endophytic fungi

*Cannabis sativa* was collected from Northeast Thailand. The samples were kept in sterile plastic zip bags and stored at 4 °C until further use following the protocol by Suebrasri et al.^29^. The plant samples were placed onto potato dextrose agar medium (PDA) containing 200 ppm chlortetracycline, then incubated at 28–30 °C until fungal hyphae appeared. Endophyte cultures were subsequently transferred onto fresh PDA until pure cultures were obtained. Then, the fungal endophytes were identified by internal transcribed spacer (ITS), translation elongation factor 1-alpha (TEF1-α), partial β -tubulin 2 (TUB2), and calmodulin (CaM)^23,45^. PCR amplifications using with ITS1F/ITS4R and EF1-728 F/EF1-986R primer were conducted under the following conditions: an initial denaturation step of 5 min at 95 °C; followed by 40 cycles of 1 min at 95 °C, 1 min at 52 °C, 90 s at 72 °C and a final extension of 10 min at 72 °C. Thermocycler conditions using primers CMD5F/CMD6R and Btub2F/Btub4R were: initial denaturation at 94 °C for 4 min; followed by 40 cycles at 94 °C for 60 s, annealing at 55 °C for 45 s, and extension at 72 °C for 1 min; and a final extension of 72 °C for 10 min. The PCR products were detected on 1.5% agarose gel and stained with ethidium bromide. The PCR products were submitted for sequencing at the First BASE Laboratories Sdn Bhd, Selangor, Malaysia. DNA sequences were analyzed using BLAST against Genbank database on the NCBI website (http://blast.ncbi.nlm.nih.gov/Blast.cgi).

### Pathogenicity tests

Because some isolated endophytic fungal species are considered as potential pathogens, a pathogenicity test was conducted to fulfill Koch’s postulates. An initial pathogenicity test was conducted in hemp leaves. Healthy hemp leaves were surface-disinfected using 70% ethanol. Inoculation was then performed using the agar plug method, as previously described by Pornsuriya et al.^21^. Each fungal isolate was cultured on PDA and incubated at room temperature for 7 days. A mycelial plug (5 mm) was cut from the colony. The hemp leaves were wounded using fine needles. Mycelial plugs were directly placed onto the hemp leaves. Each treatment consisted of 6 leaves (replicates). The inoculated samples were incubated in a humid chamber to maintain humidity (approximately 80–90%) for 7 days. The symptoms that developed on the inoculated leaves were observed and photographed daily.

### Investigation of plant growth promoting properties of endophytic fungal isolates

#### Quantification of indole acetic acid (IAA) production

The quantity of IAA produced by endophytic fungal isolates was performed using a colorimetric technique according to Salkowski’s method^46^. Five mycelium culture plugs (5 mm diameter) of each 4-day-old mycelial culture were inoculated into 100 mL of potato dextrose broth (PDB) supplemented with 0.2% of L-tryptophan. The cultures were incubated with shaking at 150 rpm, at 28 °C for 7 days under dark conditions. Then, the cell-free supernatant was retrieved by centrifugation at 4,000 rpm for 10 min. IAA analysis was carried out by mixing 1 mL supernatant with 2 mL of Salkowski’s reagent (2% 0.5 FeCl_3_ in 35% HClO_4_ solution), and then incubated at 30 °C for 30 min in the dark^47^. Total IAA production in each sample was quantified by measuring absorbance at a wavelength of 530 nm (Hitachi U-5100, Japan). The absorbance values were interpolated with a calibration curve of the standard indole-3-acetic acid for IAA quantity.

#### Quantification of gibberellic acid (GA_3_) production

The quantity of GA_3_ produced was estimated using a modified method described by Sharma et al.^48^. Five mycelium culture plugs (5 mm diameter) were inoculated into 100 mL of Czapek-Dox broth (CZ), and then incubated with shaking at 150 rpm at 28 °C for 7 days. After incubation, the cultures were centrifuged at 4000 rpm for 10 min to retrieve supernatant. Then 25 mL of the supernatant was mixed with 2 mL of 1 M zinc acetate reagent (a mixture of 21.9 g zinc acetate and 1 mL of glacial acetic acid added with distilled water to a final volume of 100 mL) in a 50 mL test tube for 2 min. Then, 2 mL of 10.6% potassium ferrocyanide was added. The mixture was subsequently centrifuged at 4000 rpm for 10 min to retrieve an aqueous phase. After that, 5 mL of the aqueous phase was mixed with an equal volume of 30% (v/v) HCl, and then incubated at 20 °C for 75 min. The absorbance of a mixture was measured at a wavelength of 254 nm using a spectrophotometer. The standard GA_3_ (Solarbio, China) was used as a standard for quantification of GA_3_ in the samples.

#### Quantification of ammonia production

Endophytic fungi were tested for ammonia production using the method described by Passari et al.^49^. Five mycelium culture plugs (5 mm diameter) of each isolate were inoculated into 100 mL peptone water in 250 mL Erlenmeyer flask. The cultures were incubated at 28 °C, 150 rpm for 7 days. After that, cell-free supernatant was retrieved by centrifugation at 4,000 rpm for 10 min. To perform ammonia production analysis, 2 mL supernatant was mixed with 0.5 mL Nessler reagent in a test tube. Ammonium concentration in each sample was determined immediately by measuring absorbance at a wavelength of 530 nm. The standard curve of (NH_4_)_2_SO_4_ was used as a standard for quantification of ammonium content in the samples.

#### Estimation of phosphate solubilization efficiency

Endophytic-fungal isolates were tested for their phosphate solubilizing ability in Pikovskaya’s medium containing 0.5% tricalcium phosphate^50^. Five agar mycelial plugs of each endophytic fungi were inoculated into 100 mL of Pikovskaya’s broth medium, and then incubated 28 °C with shaking at 150 rpm for 7 days. An uninoculated flask was set up as a control. The cultures were centrifuged at 4,000 rpm for 10 min to retrieve supernatant. The soluble phosphorus in supernatant was determined using the colorimetric molybdate blue method. The absorbance of a solution was measured at a wavelength of 820 nm using potassium dihydrogen phosphate (KH_2_PO_4_) as a standard^51^.

#### Phosphatase enzyme activity assay

The production of phosphatase enzyme by each endophytic fungal isolate was investigated in Pikovskaya’s medium containing 0.5% tricalcium phosphate. Five mycelial culture plugs (0.5 mm diameter) of a 4-day-old mycelial culture were inoculated and incubated at 28 °C with shaking at 150 rpm for 7 days. After that, the supernatant containing crude enzyme was retrieved by centrifugation at 6,000 rpm at 4 °C for 15 min. To perform phosphatase analysis, 1 mL supernatant was mixed with 4 mL sodium citrate pH 6.5 (acid phosphatase) and sodium citrate pH 8.0 (alkaline phosphatase). Then, a mixture of 1 mL of 0.025 mM pNPP and 50 µL of toluene was added, and then incubated at 37 °C for 60 min. After that, 4 mL of 0.5 M NaOH and 1 mL of 0.5 M CaCl_2_ were mixed with the supernatant solution prior to centrifugation at 10,000 rpm for 2 min. Phosphatase activity in each supernatant was determined immediately by measuring absorbance at a wavelength of 420 nm using the pNPP solution as a standard^35^.

### Preparation of fungal inocula

#### Preparation of AMF inocula

The AMF species, *Rhizophagus prolifer* PC2 − 2^6^ and *Rhizophagus aggregatus* BM−3g3^35^ were obtained from the Mycorrhiza and Mycotechnology Laboratory, Department of Microbiology, Faculty of Science, Khon Kaen University, Thailand. AMF spore production was performed according to the methods by Boonlue et al.^52^ using maize as a host plant by a pot culture technique. In short, maize seeds were surface-sterilized by applying 10% sodium hypochlorite solution for 30 min. Then, the seeds were sown into plastic pots containing twice-sterilized sandy loam soil. After that, 1 g soil of inoculum containing approximately 200 AMF spores were inoculated into each pot per 7.5 L soil as initial AMF inoculum. The maize was grown in a greenhouse with daily irrigation using tap water for 90 days. After 90 days, irrigation was stopped in order to allow the soil to dry. The dried soil was then crushed into finely ground particles. To verify the purity of spores and the total number of spores, the sucrose centrifugation method was carried out^53^. Dried soils containing AMF spores, mycelia, and colonized root fragments were then used as the inoculum for further experiment.

#### Preparation of endophytic fungi

Fungal endophyte cultures were inoculated onto PDA and incubated at 30 °C for 7 days. Then, the fungal mycelium on PDA was cut using a cork borer with a 0.5 mm-diameter. Five mycelium plugs were transferred into a fresh tube containing 30 g of sterilized sorghum seeds. The cultures were incubated under static condition for 14 days or until full colonization on sorghum seeds was observed. The seeds with full colonization of endophytic fungi were then used as inoculum in the next experiment.

### Pot experiment

#### Preparation of cannabis plants

*Cannabis sativa* subsp. *sativa* RPF3 (Hemp) was provided by the Cannabis Research Institute, Khon Kaen University, Khon Kaen, Thailand. Plant cuttings were cloned from 8-week mother plants, which were at a vegetative stage. Branches with a couple of nodes below the top were cut off. These cuttings were transferred into trays containing sterilized peat moss, and subsequently grown under a plastic dome in order to regulate transpiration levels and to keep the cuttings well hydrated. The treatment with AMF and endophytic fungi was conducted by inoculating AMF spores at a concentration of 200 spores pot^− 1^, while inoculating 2 endophytic fungi-infected sorghum seeds adjacent to the roots on the day of plant cuttings transferred into the trays. Cuttings were grown for 2 weeks at 28 °C, with 18-hour light per day using a RGB (a mixture of red, blue and green light) LED light having an intensity of 120 µmol m^− 2^ s^− 1^^6^ prior to pot trials.

#### Soil preparation

Properties of loamy sand soil used in this study were as follows: pH 5.9, electrical conductivity (EC) 0.14 dS/m, organic matter (OM) content 6.4 mg g^− 1^, available nitrogen (N) content 60 mg kg^− 1^, phosphorus (P) content 145 mg kg^− 1^, potassium (K) content 88 mg kg^− 1^, available phosphorus content 22 mg kg^− 1^, exchangeable potassium content 50 mg kg^− 1^, calcium (Ca) content 790 mg kg^− 1^, sodium (Na) content 29 mg kg^− 1^ and magnesium (Mg) content of 68 mg kg^− 1^. Rocks, wood chips, and plant debris in soil samples were removed. The soil samples were sterilized by autoclaving twice at 121 °C at a pressure of 15 psi (1.04 bar) for 120 min. Soils were then filled into 30-cm diameter pots with a total soil volume of 7.5 L per pot for the cultivation of the cuttings.

#### Pot experimental design

The experiment was conducted in enclosed greenhouses at the Cannabis Research Institute, Khon Kaen University, Khon Kaen, Thailand. The experiment was arranged as a completely randomized design (CRD), consisting of 6 treatments with 6 replications, a total of 36 pots. Six treatments were as follows: T1: control without microbial inoculum and synthetic fertilizer, T2: plants without microbial inoculum and with added synthetic chemical fertilizer (N-P-K = 15-15-15) at a ratio of 250 kg per hectare (kg ha^− 1^) as suggested by Deng et al.^54^, T3: plants inoculated with *Rhizophagus prolifer*, T4: plants inoculated with *Rhizophagus aggregatus*, T5: plants inoculated with *Lasiodiplodia theobromae*, and T6: plants inoculated with *Macrophomina phaseolina*. The T2 treatment was designed to evaluate the effectiveness of inoculation with the AMF and endophytic fungi species in enhancing plant growth and yield. Specifically, this treatment allows comparison between biological fertilization (AMF inoculation) and conventional chemical fertilizer application. The objective is to determine whether AMF can partially or fully substitute chemical fertilizers by improving nutrient uptake and plant performance.

#### The efficiency of AMF and endophytic fungi on plant growth promotion under greenhouse conditions

Cuttings inoculated with AMF or endophytic fungi were transferred into pots (30 cm diameter) containing sterilized soil. Plants were watered regularly to avoid drought. Data were collected 90 days after plantation.

#### Plant performance

Plant growth parameters including SPAD, height, trunk diameter, leaf area index, leaf dry weight, shoot dry weight, stem dry weight, root dry weight, and bast-fiber dry weight were measured at harvest. Greenness of leaves was determined from the second expanded leaf from the top of the main stem using a SPAD meter (SPAD 250 + KONICA MINOLTA, Japan). Plant height was measured by a standard stick method. Stem diameter was measured at 2.5 cm above the ground using a Vernier caliper (Mitutoyo, Japan). All fresh leaves were collected for measuring leaf area using a LI-3100 C area meter (LI-COR Bioscience, Thailand). To determine biomass, plants were dried in an oven at 80 °C until a constant weight was obtained. The bast-fibers per pot were weighed.

Fresh root samples at a ratio of 10% (by weight) of total root mass were collected for determination of root traits. Root samples were scanned using a root scanner (Epson perfection V700 photo). Dry weight and specific root length of the root samples were determined. Root tissue density was determined as the ratio of root dry weight to root volume. Data analysis was performed by the WINRHIZO Pro2004a software (REGENT Instruments Inc., QC, Canada).

#### Determination of nutrient uptake

Nutrient concentrations (nitrogen (N), phosphorus (P), potassium (K)) were measured from dried stems, leaves, roots, and bast-fibers, which were ground into powder prior to the analysis. N concentration was measured using the micro-Kjeldahl method^55^ followed by the indophenol blue method^56^. P and K concentrations were determined using the wet oxidation method with HNO_3_:HClO_4_ (2:1, v/v). Total P concentration was determined using the molybdovanadate with acid persulfate digestion method^57^, and then the absorbance of the samples was measured using a spectrophotometer at a wavelength of 420 nm. K content in the solution was determined using a flame photometer at 768 nm (Flame photometer, Model 410 Sherwood, United Kingdom)^58^.

#### Quantification of AMF spore number and root colonization by AMF and endophytic fungi

Root samples were randomly collected for determination of AMF and endophytic fungi root colonization intensity. The roots were washed with tap water three times, and then treated with 10% KOH for 5 min at 95 °C. After that, samples were neutralized by submerging in 2% HCl solution overnight. The samples were then stained with 0.05% trypan blue solution^59^. Stained roots were cut into pieces with a length of 1.0 cm each, and then arranged onto slides. The cellular structure of AMF including vesicles, arbuscules, and hyphae were observed under a microscope, and the colonization intensity was determined following the method described in Trouvelot et al.^60^. Fungal endophyte colonization was carried out in a similar manner to the AMF colonization assessment. The root colonization frequency of endophytic fungi was determined according to the method described by Mehmood et al.^61^.

### The efficiency of AMF and endophytic fungi on fiber quality of hemp and cannabinoid contents

#### Chemical composition analysis of fibers

Plants at harvest were cut to remove bast fibers. Bast fibers were oven-dried at 70 °C for 48 h, ground, and finally sifted through a 1-mm pore diameter sieve prior to analysis. Acid detergent fiber (ADF) and neutral detergent fiber (NDF) analyses were carried out according to AOAV^62^ and Van Soest et al.^63^ using an Ankom 200^®^ fiber analyzer. Acid detergent lignin (ADL) analysis was conducted using the sulfuric acid dilution method^64^. Hemicellulose and cellulose contents were estimated according to the following formulas: Hemicellulose = NDF-ADL; Cellulose = ADF-ADL; Lignin = ADL.

#### Extraction and quantification of cannabinoids

Leaf and shoot samples (approximately 5 g each) were ground into powder and then heated at 100 °C for 3 h prior to the extraction of cannabinoids. The samples were mixed with hexane at a ratio of hexane (mL): sample (g) = 10:1 v/w, and incubated at 4 °C for 24 h. The solvent layer was separated and filtered through a vacuum funnel (Buchner funnel, Fisher Scientific, Sweden). The solvent was evaporated using a rotary evaporator (RE300/MS Rotary Evaporator, TEquipment, United States) to retrieve crude extract. Cannabinoid content in the crude extract samples was analyzed using high-performance liquid chromatography (HPLC Alliance system waters and Column CORTECS Shield, Agilent Germany)^6^.

#### HPLC analysis of the extracted cannabinoids

Types of cannabinoid compounds were determined by comparison with the retention times of the standard compounds including Cannabidiol (CBD), Tetrahydrocannabinol (THC), Cannabinol (CBN), Cannabidiolic acid (CBDA), Tetrahydrocannabinolic acid (THCA), and Cannabigerol (CBG). Quantification of cannabinoid compounds was carried out by interpolating peak areas of the target compounds with the calibration curves of the corresponding compounds^6^. The mobile phase was a mixture of acetonitrile and 0.1% triethylamine (TFA), diluted using distilled water at a mixture: water ratio of 40:60 v/v, and adjusted to pH 2.2. Prior to use, the mobile phase solution was filtered through a hydrophilic PTFE membrane filter (0.5 μm, ADVANTEC, Toyo Roshi Kaisha, Ltd. Tokyo, Japan). The HPLC condition was as follows: flow rate 1.5 mL min^− 1^; column temperature 35 °C; monitoring at a wavelength of 228 nm using a UV absorbance detector; column, CORTECS Shield RP18 (2.7 μm particle size, 4.6 mm × 150 mm).

### Statistical analysis

Data were analyzed using the Statistix 10 software. Analysis of variance (ANOVA) was performed to analyze differences among the means of the data. Fisher’s Least Significant Difference (LSD) was applied to analyze significant differences among data at a 95% confidence interval (*P* ≤ 0.05).

## Data Availability

The original data can be obtained from the last author upon reasonable request.
